# Subcutaneous and extravascular implantable cardioverter-defibrillators: a clinical framework for breast surgery, pregnancy, lactation, radiation therapy, and breast imaging

**DOI:** 10.1093/europace/euag274

**Published:** 2026-09-29

**Authors:** Kamala P Tamirisa, Ian Crozier, Lucas Boersma, Jodie L Hurwitz, Amin Al-Ahmad, Mark S Link, Miral Amin, Devika Adusumilli, Luigi DiBiase, Sana M Al-Khatib, Andrea M Russo

**Affiliations:** Division of Cardiology, University of Texas Southwestern Medical Center, 2001 Inwood Road, Dallas TX 75390, USA; Department of Cardiology, Christchurch Hospital, Christchurch, New Zealand; Cardiology Department, St. Antonius Hospital Nieuwegein/Amsterdam University Medical Center, Nieuwegein & Amsterdam, Netherlands; North Texas Heart Center, Dallas, TX, USA; Texas Cardiac Arrhythmia Research Institute, Austin, TX, USA; Division of Cardiology, University of Texas Southwestern Medical Center, 2001 Inwood Road, Dallas TX 75390, USA; Surgical Breast Oncology and Oncoplastic Breast Surgery, Venus Breast Center, Southlake, TX, USA; Department of Internal Medicine, Medical City Hospital, Fort Worth, TX, USA; Division of Cardiology, Montefiore Medical Center, Bronx, NY, USA; Division of Cardiology, Duke University Medical Center, Durham, NC, USA; Division of Cardiology, Cooper Medical School of Rowan University, Camden, NJ, USA

**Keywords:** Breast surgery and ICDs, Subcutaneous ICD, Extravascular ICD, Women and CIEDs

## Abstract

The subcutaneous implantable cardioverter-defibrillator and extravascular implantable cardioverter-defibrillator do not require intravascular, intracardiac, or epicardial leads, making them attractive alternatives to transvenous implantable cardioverter-defibrillators in patients without bradycardia pacing or cardiac resynchronization therapy indications. These systems are especially useful for patients with venous access challenges and those at risk for endovascular infections, including patients with renal failure or those who are immunocompromised. Women with prior or anticipated breast surgery, such as mastectomy or breast reconstruction surgery, or those who are lactating present unique anatomic, procedural, and post-operative quality-of-life considerations. This commentary provides a practical, scenario-based framework for managing this unique patient group. Key considerations include individualized incision and pulse generator planning, proactive multidisciplinary coordination, procedure-specific management, and shared decision-making in patients with prior or anticipated breast augmentation or reduction, pregnancy, lactation, mastectomy, radiation therapy, and breast imaging.

## Graphical Abstract

**Graphical Abstract:**
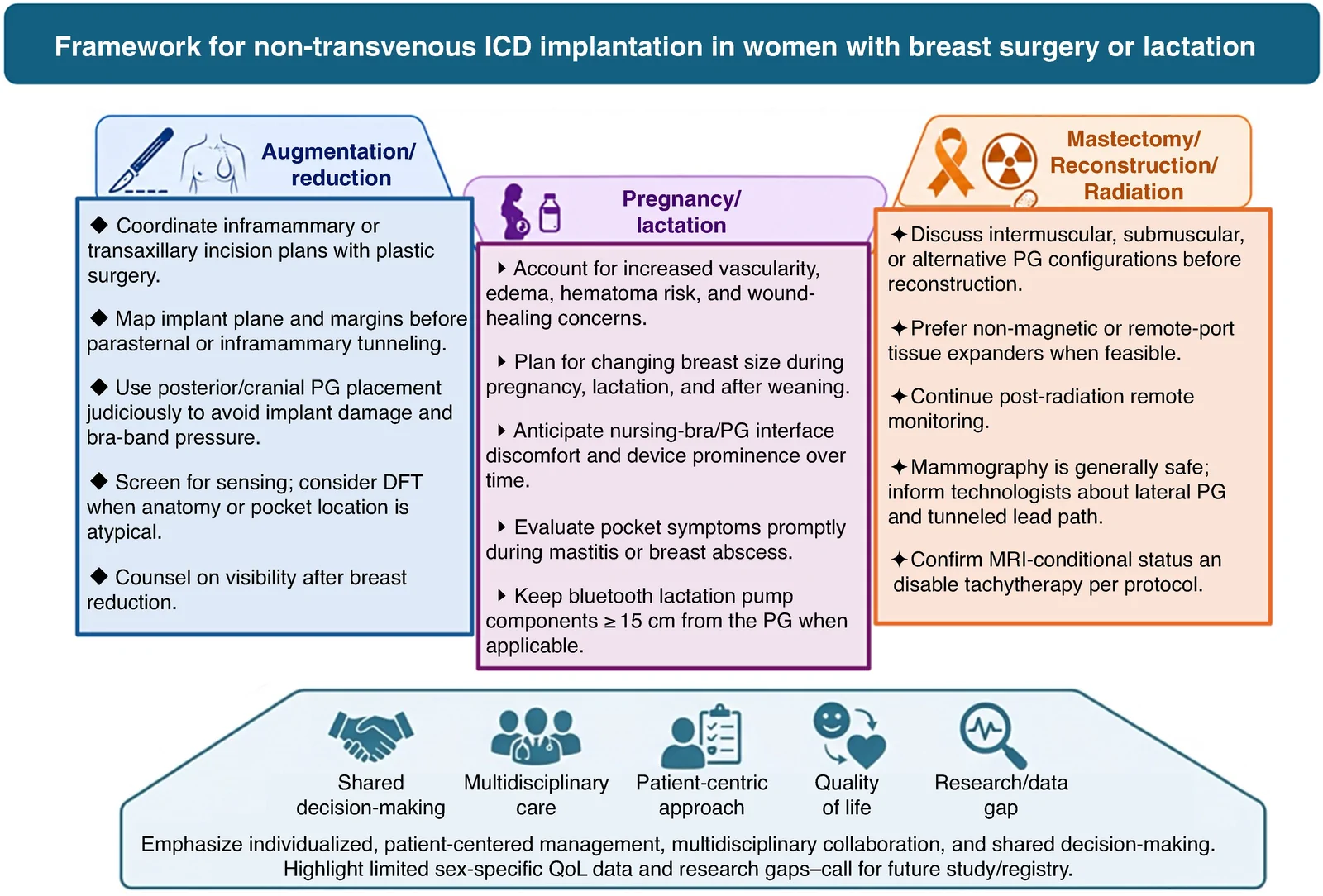
Practical framework for non-TV-ICD management in women with breast surgery, pregnancy, lactation, radiation therapy, and breast imaging.

## Background

The subcutaneous implantable cardioverter-defibrillator (S-ICD) and extravascular implantable cardioverter-defibrillator (EV-ICD) eliminate the need for intracardiac, intravascular, or epicardial lead placement, offering comparable safety and efficacy to transvenous implantable cardioverter-defibrillators (TV-ICDs).^1–4^ This makes them particularly useful for younger patients, as these individuals have a long-anticipated device exposure and are at increased risk for long-term lead failure.^5,6^ Subcutaneous implantable cardioverter-defibrillator therapy is also an important option for paediatric and young patients with congenital heart disease who do not have pacing indications.^7^  *Table 1* summarizes the key features of TV-ICD, S-ICD, and EV-ICD systems. Both S-ICD and EV-ICD are approved for patients not requiring bradycardia pacing or cardiac resynchronization therapy (CRT).^13,14^ The MODULAR Anti-Tachycardia Pacing (ATP) trial showed promising results with the S-ICD in wireless communication with a leadless pacemaker as a viable alternative to a single-chamber TV-ICD system but has not yet been commercialized.^15^ Another extravascular, intercostal ICD with pectoral or mid-axillary pulse generator (PG) placement is under development with limited data available.^16,17^ The current paper focuses on the S-ICD and EV-ICD systems (referred to as ‘non-TV-ICD’ for this paper) and addresses the unique anatomic, procedural, and quality-of-life (QoL) considerations for non-TV-ICD implantation in women with breast surgery or lactating breast tissue, offering a scenario-based framework to guide shared decision-making (SDM) and multidisciplinary care.

**Table 1:** Key features of TV-ICD, S-ICD, and EV-ICD

Feature	TV-ICD (varied manufacturers)	S-ICD (Boston Scientific)	EV-ICD (Medtronic)
Lead placement	Intracardiac	Subcutaneous, parasternal	Subcutaneous, substernal
PG location	Subclavian	Left mid-axillary (intermuscular or submuscular)	Left mid-axillary (above serratus anterior)
PG volume	∼30 cm^3^	∼60 cm^3^	∼33 cm^3^
Defibrillation energy^a^	Up to 35–41 J (median DFT ∼ 10–15 J)^8,9^	Up to 80 J (median DFT ∼ 36 J)^10^	Up to 40 J (median DFT ∼ 15 J)^6^
Anti-tachycardia pacing	Yes	No	Yes
Bradycardia pacing	Full range	Post-shock only (30 s) Compatible with bipolar pacemaker	Pause-prevention only (programmable 5–15 s, switches from OVO mode to VVI 40 beats per minute for 30 s, and switches back to OVO after 30 s) Compatible with bipolar pacemaker
CRT capability	Yes	No	No
Projected battery longevity^a^	∼10–14 years	∼7 years	∼10–12 years
Vascular access required	Yes	No	No
MRI-conditional	Yes	Yes	Yes
Prior sternotomy	Compatible	Compatible	Off label
Key advantage in breast surgery patients	Subclavian PG requiring vascular access and in radiation exposure area	Avoids vascular access; PG away from traditional radiation exposure area	Avoids vascular access, PG away from traditional radiation exposure area Smaller PG

^a^Projected battery longevity values are approximate and model/manufacturer specific and should not be interpreted as fixed service-life estimates. Actual longevity depends on device programming, pacing burden, capacitor charges, and the frequency of delivered or diverted therapies, including shocks for ventricular arrhythmias.^11,12^

CRT, cardiac resynchronization therapy; DFT, defibrillation threshold; EV-ICD, extravascular implantable cardioverter-defibrillator; PG, pulse generator; S-ICD, subcutaneous implantable cardioverter-defibrillator; TV-ICD, transvenous implantable cardioverter-defibrillator.

### Breast anatomy considerations

The currently available non-TV-ICDs require two incisions: one typically at the lower end of the sternum for lead placement, which overlaps with the medial border of the breast, and the second at the left mid-axillary region for PG placement on the lateral chest wall between the 5th and 6th intercostal spaces, which overlaps with the lateral border of the breast (*Figure 1*). The PG is anchored to the underlying fascia or secured within an intermuscular pocket, and the lead is secured via a suture sleeve with nonabsorbable suture to prevent migration.^18^ Pulse generator migration is recognized as a potential complication for both TV and non-TV-ICDs, with female sex identified as a significant predictor of device movement and discomfort (FDA’s Manufacturer and User Facility Device Experience analysis: 137 migration reports among over 15 000 S-ICDs).^19^ The lateral PG position interferes with the bra band in most cases.^20^ The EV-ICD PG is ∼50% smaller than the S-ICD and is positioned above the serratus anterior, whereas the S-ICD PG is most often placed in an intermuscular location between the serratus anterior and the latissimus dorsi.^17,21^ The position of the S-ICD PG may be customized relative to the bra band to improve tolerance while maintaining a posterior position and considering the PRAETORIAN SCORE to ensure adequate defibrillation efficacy.^6,22^

**Figure 1:**
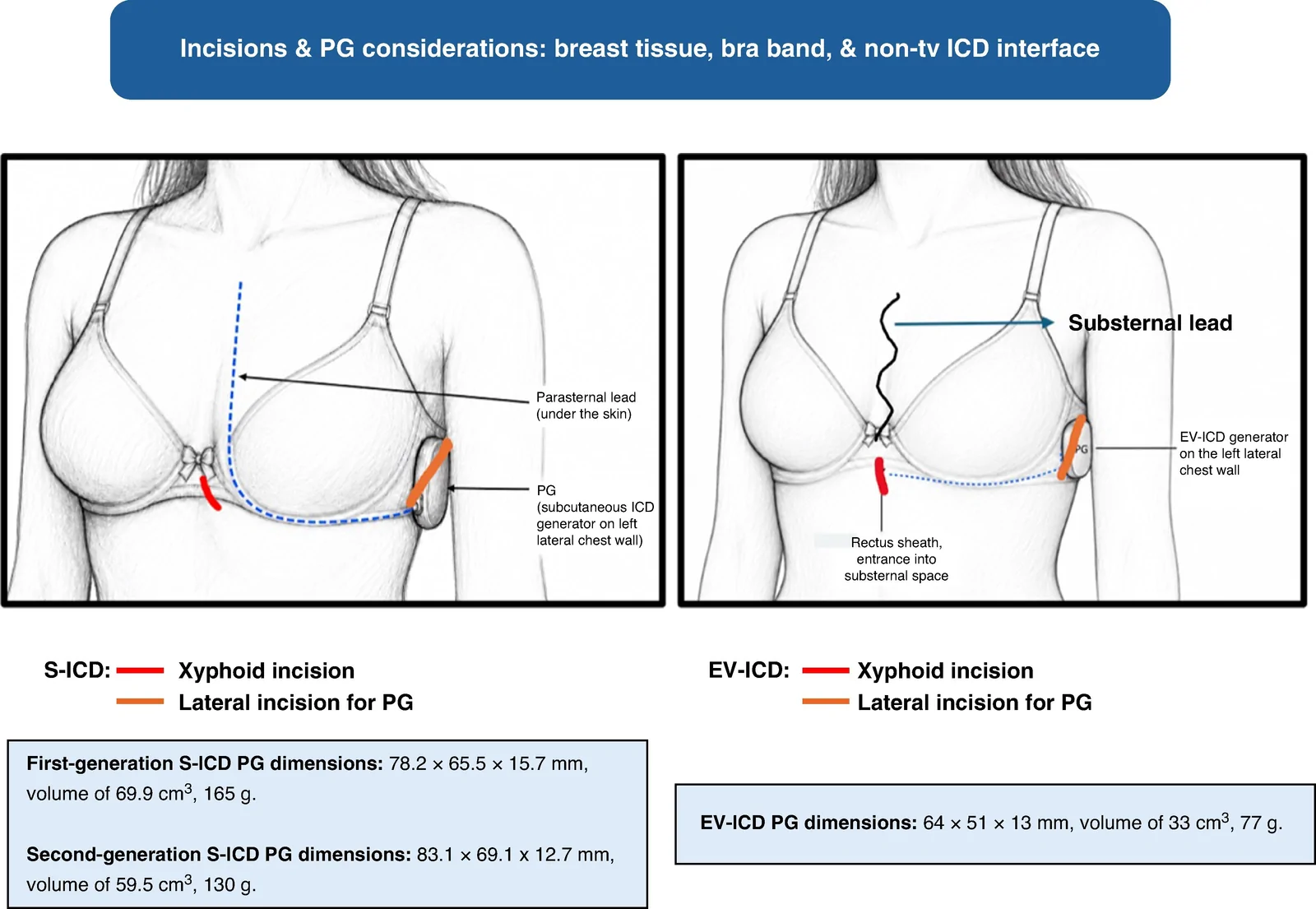
Incisions and PG considerations: breast tissue, bra band, and non-TV-ICD interface. PG, pulse generator; TV-ICD, transvenous implantable cardioverter-defibrillator.

Women who have had or need breast surgery, such as augmentation, mastectomy, reconstruction, or those with lactating breast tissue, present unique considerations regarding non-TV-ICD implantation. Key technical factors include tunnelling leads through the inframammary (along the inferior margin of the breast) and parasternal areas and optimal PG positioning along the lateral chest wall. Variations in body habitus and breast size may further influence tissue depth and appropriate PG placement. Cosmetic concerns surrounding PG size and discomfort, PG migration, and interference with bra lines have been reported, playing an important role in SDM.^17,23^ Pre-procedure planning and individualized assessment are therefore essential for optimizing acute and long-term comfort and cosmetic outcomes. The lack of specific clinical guidance for non-TV-ICD implantation in women with altered breast anatomy underscores the need for a practical framework (*Graphical Abstract*) to guide peri-procedural planning in this population.

### Impact of non-transvenous implantable cardioverter-defibrillators on quality of life

Data on the impact of non-TV-ICDs on QoL remain limited. In a single-centre retrospective women-only study (94% intermuscular position; 52% with newest generation S-ICD), postoperative pain was reported in over 50% and chronic discomfort in 44% of women, especially related to bra-edge or underwire contact, highlighting the importance of individualized patient-guided pre-procedure planning.^21^ Other issues included discomfort during intimate contact (29%), awareness of the device, and 13% of women urging implanters to consider bra position, with 63% preferring a smaller PG design.^21^ In the S-ICD Post-Approval Study, overall device-related complications, including discomfort, device movement, haematoma, and inadequate incision healing, were higher in women compared with men (15.7% vs. 10.4%; *P* = 0.0023).^4^ However, in a propensity-matched, multicentre international analysis from the i-SUSI project, women were less likely to experience appropriate S-ICD therapy, while no significant sex differences were observed in device-related complications or inappropriate shocks.^24^ In a secondary analysis of the PRAETORIAN trial [426 patients with S-ICD and 423 patients with TV-ICD; with only 19–20% women in both S-ICD (84 women) and TV-ICD (76 women) groups], there was no difference in QoL between S-ICD and TV-ICD patients despite the larger PG size of the S-ICD. No differences were observed between men and women, except in those with a BMI of <25, where TV-ICD implantation was associated with better mental health (Short Form Health Survey-36).^25^ Similarly, patient-reported outcomes (PRO) following S-ICD implantation were assessed using the Florida Patient Acceptance Survey by the ‘S-ICD Rhythm Detect’ investigators.^26^ Their analysis [176 patients, 22% women (39 women)] revealed positive patient acceptance of the S-ICD, even in patients with thinner body habitus, suggesting no sex-based differences. In the MODULAR ATP study, S-ICDs were associated with better positive appraisal (how favourably a patient perceives the device and would choose to receive it again) than TV-ICDs [positive appraisal score of 88 (IQR: 75–100) vs. 81 (IQR: 63–94), *P* = 0.02].^16^ Together, these data suggest that overall patient acceptance is generally comparable between S-ICDs and TV-ICDs. However, given the low proportion of women in these trials and the lack of prospective women-specific QoL data, the results should be interpreted cautiously.

Sears *et al*.^27^ evaluated PRO (12-Item Short Form Survey, including mental and physical health QoL scores at baseline and at 6 months) with the EV-ICD in a prospective, single-arm, non-randomized, premarket approval study of 247 patients. Although EV-ICD patients reported better physical QoL than S-ICDs, the comparison was indirect using historical S-ICD and TV-ICD cohorts, and the results should be interpreted cautiously, as early-implanter bias, new technology bias, and study-era effects may explain these differences rather than device type alone.

Data regarding ICD choice and QoL remain limited in women, highlighting the need for greater enrolment of women in device studies to inform device-related QoL assessments, especially in those with altered breast anatomy.

### Clinical scenarios

The key clinical considerations for each scenario are summarized in the *Graphical Abstract*.

#### Breast augmentation and reduction

Inframammary incisions used for breast augmentation and reduction may overlap with the tunnelled lead path of non-TV-ICDs, requiring coordination with the surgical team. Complications reported with TV-ICDs include breast implant rupture from mechanical stress of PG proximity and PG migration, especially with older breast implants.^28,29^ While these specific complications have not yet been reported with S-ICDs or EV-ICDs, this is a possibility given the anatomic proximity to breast implants and should be considered during SDM.

Transaxillary incisions, used for breast augmentation in women who prefer no visible breast scar, may interfere with the optimal PG pocket location and potentially increase the risk of left arm myopotential oversensing, especially if the EV-ICD PG is positioned more cranially.^30^ Subglandular breast implants may overlap with the subcutaneous plane used for parasternal lead tunnelling with S-ICDs, warranting careful identification of implant margins during tunnelling. For subpectoral S-ICD implants, incision planning, posterior positioning of the PG away from the implant, and the use of fluoroscopy to plan the left lateral incision are important to avoid breast implant damage. Cosmetic surgeons should also exercise caution when planning breast augmentation in the setting of pre-existing or planned non-TV-ICDs.

Given the heterogeneity of breast augmentation scenarios, management should be individualized. Multidisciplinary collaboration between electrophysiology and plastic surgery is important, especially in cases with prior revisions and scarring.^18^ Standard intra-operative device management, including bipolar electrosurgical utilization, and tachytherapy deactivation should be applied, with the understanding that S-ICDs and EV-ICDs do not provide bradycardia-pacing support.^19^

Breast reduction may result in increased visibility of the parasternal lead and PG due to reduced soft-tissue coverage, with potential cosmetic and comfort implications. However, the absence of a PG in the subclavian region may offer an advantage with a well-planned non-TV-ICD placement.

#### Pregnant and lactating breast tissue

During pregnancy and lactation, breast vascularity and tissue oedema increase, and non-cardiovascular implantable electronic device (CIED) surgical literature reports higher rates of bleeding, haematoma, wound dehiscence, and infection.^31^ Lead tunnelling and PG placement with S-ICDs and EV-ICDs adjacent to breast tissue planes raise similar theoretical concerns and call for thoughtful procedural timing, if clinically feasible. If implantation cannot be deferred during pregnancy, fluoroscopy requirements differ by system: EV-ICD implantation requires fluoroscopic guidance, whereas S-ICD implantation is primarily guided by anatomical landmarks and can usually be performed without fluoroscopy.^2,6,14^ When fluoroscopy is required, radiation exposure should be minimized according to as low as reasonably achievable principles.^32^

Lactation mastitis may increase the risk of secondary bacterial infection, and cases requiring device extraction have been reported with transvenous pacemakers.^33^ With S-ICDs and EV-ICDs, there is a lower risk of bacteraemia from device infection, and infection-related management is simpler than with TV-ICDs, as it does not require transvenous lead extraction.^34^ Changes in breast size during lactation and after weaning may alter the perceived prominence or position of the PG, cause discomfort, and affect the nursing bra band–PG interface. Women may not return to their pre-pregnancy BMI and may benefit from proactive anticipatory guidance from the care team, addressing both the physical and psychological aspects of devices during and after pregnancy.

Lactation pumps are generally considered safe in patients with non-TV-ICDs if no Bluetooth is utilized.^35^ If Bluetooth is used, a distance of at least 15 cm (5.9 inches) between the transmitter/receiver and the PG is recommended.^22^ There are no published studies on lactating breast tissue–CIED interactions.

Prospective data on the clinical and PRO of women with S-ICDs and EV-ICDs during pregnancy and lactation will be important to better counsel women of childbearing age who are candidates for non-TV-ICD therapy.

#### Mastectomy, axillary surgery, reconstruction, and radiation therapy

Non-TV-ICDs offer advantages in breast cancer patients, including avoidance of vascular access and lower infection risk, particularly in those with prior lymph node dissection and radiation exposure.^36,37^

Mastectomy may create a thin anterior chest wall soft-tissue envelope at the mid-axillary line, especially in thin patients or after modified radical mastectomy, leading to erosion, lead visibility, and delayed wound healing, as has been described with ICDs and pacemakers.^38^ Acellular dermal matrix and dermal grafts, used for erosion prevention with transvenous CIEDs, may be considered for non-TV-ICDs in selected cases, although supporting evidence is lacking.^22^ The use of a latissimus dorsi flap solely to prevent erosion may not be viable with an intermuscular S-ICD PG, as it may disrupt the intermuscular plane.^39^ In these cases, alternative strategies, such as abdominal or distant autologous flaps, or TV-ICDs may be considered.^40^

In patients with a pre-existing non-TV-ICD requiring sentinel lymph node biopsy or axillary lymph node dissection, direct instrumentation or needle passage near the PG should be avoided.^27^ In patients requiring non-TV-ICD placement after prior axillary surgery, lateral chest wall scarring and fibrosis may potentially complicate intermuscular pocket creation, warranting intra-operative assessment of tissue planes. No published data exist on the interaction between axillary surgery and non-TV-ICDs. Surgical drain placement post-mastectomy or axillary dissection may be in close proximity to the lateral PG pocket, warranting careful positioning and consideration of antibiotic prophylaxis.^27^

Reconstruction using a latissimus dorsi myocutaneous flap may disrupt the lateral PG pocket, and abdominal-based free flaps or PG repositioning should be considered.^19,41^ Conversely, in patients requiring non-TV-ICD after prior latissimus dorsi flap reconstruction, the intramuscular plane may no longer be available, necessitating a submuscular approach or alternate device system.^42,43^ Other procedures involving the thoracodorsal pedicle, such as vascularized lymph node transfer, require similar anatomic consideration relative to the PG pocket.^44^

Breast tissue expanders with magnetic ports, used temporarily for reconstruction, pose an additional concern for unintended tachytherapy inhibition. Khan *et al.* reported a case in which the magnetic port of a breast tissue expander triggered ICD magnet-mode behaviour, temporarily suspending tachyarrhythmia detection and therapy (reported with TV-ICDs). Because this interaction may be position dependent and may be missed during routine device evaluation, non-magnetic expanders should be considered in patients with ICDs, and prompt interrogation of any unexpected device alerts should be prioritized.^9,25,45^

Radiation therapy to the thorax may pose additional challenges. Radiation-induced fibrosis may alter intermuscular pocket integrity, and capsular contracture around the PG may occur, as documented around breast implants, though data are largely unknown.^46,47^ Neutron-producing radiation, including ≥10 MV photon beams and proton therapy, is the principal risk factor for CIED malfunction, including temporary device memory single event upset resulting in inappropriate shock, with an ∼10-fold increased risk compared with non-neutron radiation.^48^ This is of particular concern for TV-ICDs with subclavian PGs located in conventional radiation fields. In contrast, the lateral chest wall PG position with S-ICDs and EV-ICDs is typically outside standard breast chest wall-only radiation fields, potentially minimizing PG radiation exposure. However, Tajstra *et al.*^49^ reported major battery depletion in explanted S-ICDs after stereotactic radiotherapy in an *in vitro* study, highlighting the need for further data collection and close monitoring for radiation-induced damage. CIED relocation has been reported with TV-ICDs, but PG relocation might not be feasible in all cases, and in such cases, the PG may be left in place and subsequently replaced (if damaged during radiation therapy) with a wearable defibrillator used as a bridge, if clinically indicated.^1,50^ Post-radiation monitoring in collaboration with the radiation oncology team is important, and remote monitoring may be particularly useful, especially for patients distant from the implanting centre [47]. Emerging modular systems combining leadless pacemakers with S-ICDs may introduce additional considerations in this population, though specific data are not yet available.

#### Mammography considerations

Currently available expert consensus supports the safe use of mammography in patients with CIEDs, including S-ICDs and EV-ICDs.^19^ While the parasternal lead with S-ICDs may cause discomfort during compression, the lateral PG position typically does not interfere with mammographic image acquisition or interpretation. There have been no reports of device malfunction associated with mammography. However, there may be a theoretical risk for lead dislodgement with excessive manual compression in patients with S-ICDs, although data are lacking.^1^ Patients should inform technologists about the lateral PGs (instead of subclavian TV-ICDs) or tunnelled lead paths (inframammary and parasternal), so that excessive compression over these areas can be avoided. Breast ultrasound imaging is safe, but MRI scans should be preceded by confirming that the device is MRI conditional and programmed to MRI-safe mode, with tachycardia therapies temporarily disabled to avoid inappropriate shocks.^10,51^

The key multidisciplinary considerations across breast-related clinical scenarios are summarized in *Table 2*. A stepwise approach for the evaluation of a newly detected breast mass in patients with non-TV-ICDs is provided in *Table 3*.

**Table 2:** Multidisciplinary quick-reference framework for S-ICD and EV-ICD across breast-related clinical scenarios

Clinical scenario	Multidisciplinary team	Key considerations	Practical device-related management
Breast augmentation/reduction	Plastic surgery Breast surgery	Coordinate inframammary or transaxillary incision plans with EP. Map implant plane and margins before tunnelling. Reduction may increase lead/PG visibility.	Subpectoral/subglandular implant planes may overlap S-ICD tunnelling. Transaxillary incisions may affect PG pocket and myopotential oversensing.
Breast imaging/screening	Radiology Imagers Gynaecology Oncology Primary care	Mammography and ultrasound are safe. Be aware of the lateral PG and tunnelled lead (S-ICD), so excessive compression over hardware is avoided. Confirm MRI-conditional status, MRI-mode programming with tachytherapy suspended, and restored afterwards.	Parasternal S-ICD lead may cause compression discomfort.
During pregnancy	Obstetrics Gynaecology	Anticipate increased breast vascularity and tissue oedema. Time any elective device/breast procedure when clinically feasible. Consider PG prominence and comfort as breast anatomy changes. For EMI-generating procedures/cautery, use standard peri-procedural ICD management.	S-ICDs can be done with minimal or zero fluoroscopy. EV-ICDs need fluoroscopy for implantation.
Lactation-related	Gynaecology Lactation expert Primary care	Assess breast infection promptly given mastitis-related secondary infection risk. Non-TV-ICDs carry lower bacteraemia risk and simpler infection management than TV-ICDs (no lead extraction). Avoid prolonged pressure over the PG. Reassess comfort after weaning.	Keep Bluetooth breast-pump components ≥ 15 cm from the PG.
Mastectomy/axillary surgery	Surgical Oncology Breast surgery Plastic surgery	Avoid needle passage and direct instrumentation near the PG or lead. Position drains away from the lateral PG pocket. Consider antibiotic prophylaxis. Monitor thin post-mastectomy tissue for erosion and impaired healing.	Prior axillary scarring/fibrosis may complicate future pocket creation.
Reconstruction/tissue expander	Plastic surgery	A latissimus dorsi flap may disrupt the lateral/intermuscular PG pocket-favour abdominal/distant autologous flaps or PG repositioning. Prefer non-magnetic (remote-port) tissue expanders.	Intermuscular S-ICD pocket is specifically vulnerable to latissimus flap disruption. Magnetic expander port can trigger magnet mode and suspend therapy.
Radiation therapy	Radiation oncology	Identify the PG at treatment planning and minimize direct exposure. Establish device monitoring during/after treatment (remote monitoring useful).	Neutron-producing radiation (≥10 MV photons, protons) is the principal malfunction risk (∼10-fold). Lateral non-TV PG is typically outside standard breast/chest-wall fields.
Any procedure with electrosurgery/EMI	All proceduralists	Suspend tachytherapies. Use bipolar electrosurgery when feasible. Keep external defibrillation available. Reactivate therapies before discharge.	Non-TV-ICDs do not deliver bradycardia pacing, so deactivation carries no pacing-loss concern.

EMI, electromagnetic interference; EP, electrophysiology team; EV-ICD, extravascular implantable cardioverter-defibrillator; PG, pulse generator; S-ICD, subcutaneous implantable cardioverter-defibrillator; TV-ICDs, transvenous implantable cardioverter-defibrillators.

**Table 3:** Practical guidance for non-EP clinicians evaluating a newly detected breast mass in patients with S-ICDs and EV-ICDs

Step	Practical action	Device-specific considerations and management
1. Identify device	Confirm S-ICD vs. EV-ICD, obtain device card, document PG location and lead course	S-ICD: parasternal subcutaneous lead; EV-ICD: substernal lead; neither provides bradycardia pacing
2. Imaging	Proceed with indicated breast imaging	Mammography and ultrasound are safe; inform technologist of PG/lead location and avoid excessive compression over hardware. Confirm MRI-conditional status before MRI
3. Biopsy	Biopsy as clinically indicated	Core biopsy is generally safe; avoid needle passage or instrumentation directly over or near PG or parasternal lead
4. Surgical planning	Refer to EP before surgery, reconstruction, or procedures involving EMI	Plan around device location; for electrosurgery, suspend tachytherapies as indicated and ensure external defibrillation availability
5. Cancer treatment	Coordinate axillary surgery, reconstruction, and radiation planning with EP input	Avoid direct instrumentation near hardware; protect PG pocket; establish device monitoring during/after radiation

EMI, electromagnetic interference; EP, electrophysiology team; EV-ICD, extravascular implantable cardioverter-defibrillator; PG, pulse generator; S-ICD, subcutaneous implantable cardioverter-defibrillator.

### Gaps and future directions

S-ICDs and EV-ICDs are increasingly being implanted in patients, offering important benefits as fully extravascular technologies. However, significant gaps remain in understanding their long-term management in women with altered breast anatomy or during pregnancy, lactation, radiation therapy, and imaging. Sex-specific and breast surgery-specific data and outcomes remain largely underexplored. Whether men who have undergone breast surgery have the same issues as females is not clear. As these technologies continue to evolve, and modular systems (EMPOWER—S-ICD with leadless pacemaker) become available, additional considerations may arise in this population. Device design and proof-of-concept assessments should incorporate patient perspectives, preferences, and SDM. Future studies must reflect real-world, multidisciplinary care and include long-term safety, efficacy, and patient-reported QoL outcomes. Collaboration among electrophysiologists, plastic surgeons, breast surgeons, radiation oncologists, and clinical engineers is crucial to address practical challenges and inform solutions. A lifespan-oriented, patient-centred approach is essential for the implementation of these life-saving technologies in women and should incorporate SDM from pre-procedural planning through long-term post-procedural care. A registry or multicentre study of women with prior breast surgery or radiation could help capture PRO and clinical outcomes, providing critical data to guide peri-procedural SDM and management.

## Conclusion

The management of S-ICDs and EV-ICDs in women with altered breast anatomy or during lactation, radiation therapy, and imaging requires individualized, scenario-specific strategies with attention to QoL outcomes, SDM, and multidisciplinary collaboration. Future development of these technologies should be patient centric and informed by prospective, sex-specific, and PRO research.

## Supplementary Material

euag274_Supplementary_Data

## Data Availability

The data underlying this article are available in the article and in its online Supplementary material.
