# Supplementary material for: Subcutaneous and extravascular implantable cardioverter-defibrillators: a clinical framework for breast surgery, pregnancy, lactation, radiation therapy, and breast imaging
Source: Europace. 2026 Sep 29;28(10):euag274. doi: 10.1093/europace/euag274 (PMC13685625; doi:10.1093/europace/euag274)
Supplement: Supplementary file 1 [file euag274_supplementary_data.docx]

# Your S-ICD or EV-ICD and Breast Health

Information for patients: breast procedures, imaging, pregnancy, lactation, and shared decision-making

| Know your device | Your S-ICD or EV-ICD is a non-transvenous ICD. The pulse generator (device battery) and lead are located outside the heart and blood vessels. Because the device is near breast and chest wall tissues, your heart and breast care teams should plan together. |
| --- | --- |
| Before breast procedures | Tell your breast surgeon, plastic surgeon, and other clinicians that you have an ICD. Ask where your pulse generator is located, where your lead travels, and whether your planned procedure may affect your device. |
| Breast imaging | Mammography and ultrasound are generally safe. Tell the imaging team where your device is located. MRI requires confirmation that your complete system is MRI-conditional and appropriate device programming. |
| Surgery and reconstruction | If you are having augmentation, reduction, mastectomy, lymph node surgery, or reconstruction, discuss device location before surgery. Incisions, drains, tissue expanders, and reconstruction plans may need to account for your ICD pocket and lead. |
| Comfort, clothing, & quality of life | Some patients notice pressure from bra bands or underwire bras, awareness of the device, or changes after pregnancy, weight changes, or breast surgery. Tell your care team if your ICD affects comfort, clothing choices, exercise, sleep, or daily activities. |
| Pregnancy and lactation | Discuss pregnancy planning with your cardiology and obstetric teams. Breast changes may affect comfort. During lactation, report infection symptoms promptly, discuss the breast pump details, and avoid prolonged pressure over the device. |
| Radiation therapy | Tell your radiation oncology team about your ICD. Your device location should be considered during treatment planning, and device monitoring may be needed during and after treatment. |
| Questions to ask your care team | ☐ Where is my ICD located? ☐ Where does my lead travel? ☐ Should my electrophysiology team be involved? ☐ What options can improve my comfort and quality of life? ☐ How can we make decisions that fit my goals? |

**Remember:** You are an important member of your healthcare team. Shared decision-making includes your medical needs, comfort, preferences, and quality of life.
